# Analysis of interval‐grouped data in weed science: The binnednp Rcpp package

**DOI:** 10.1002/ece3.5448

**Published:** 2019-09-13

**Authors:** Daniel Barreiro‐Ures, Mario Francisco‐Fernández, Ricardo Cao, Basilio B. Fraguela, Ramón Doallo, José Luis González‐Andújar, Miguel Reyes

**Affiliations:** ^1^ Research Group MODES, CITIC, Departamento de Matemáticas, Facultade de Informática Universidade da Coruña A Coruña Spain; ^2^ Research Group GAC, CITIC, Departamento de Ingeniería de Computadores, Facultade de Informática Universidade da Coruña A Coruña Spain; ^3^ Instituto de Agricultura Sostenible (CSIC) Córdoba Spain; ^4^ Departamento de Actuaría, Física y Matemáticas Universidad de las Américas‐Puebla Puebla México

**Keywords:** bandwidth selection, hydrothermal time, nonparametric kernel estimation, weed emergence model

## Abstract

1. Weed scientists are usually interested in the study of the distribution and density functions of the random variable that relates weed emergence with environmental indices like the hydrothermal time (HTT). However, in many situations, experimental data are presented in a grouped way and, therefore, the standard nonparametric kernel estimators cannot be computed.
2. Kernel estimators for the density and distribution functions for interval‐grouped data, as well as bootstrap confidence bands for these functions, have been proposed and implemented in the binnednp package. Analysis with different treatments can also be performed using a bootstrap approach and a Cramér‐von Mises type distance. Several bandwidth selection procedures were also implemented. This package also allows to estimate different emergence indices that measure the shape of the data distribution. The values of these indices are useful for the selection of the soil depth at which HTT should be measured which, in turn, would maximize the predictive power of the proposed methods.
3. This paper presents the functions of the package and provides an example using an emergence data set of *Avena sterilis* (wild oat).
4. The binnednp package provides investigators with a unique set of tools allowing the weed science research community to analyze interval‐grouped data.

## Graphical abstract

An R package, called binnednp, implementing nonparametric methods for interval‐grouped data is presented. In particular, weed emergence data are usually presented in a grouped way and can be analyzed using this package. An example of emergence data set of *Avena sterilis* (wild oat) is also included in the paper.

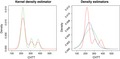

## 1 INTRODUCTION

The knowledge of the factors affecting the emergence patterns of weeds is not only interesting from a plant ecology perspective, but also in applied research, where the emergence of weeds is an important phase of the population dynamics (González‐Andújar, 2008). This critical phase has important implications, either because of its effects on the determination of competition with the crop or because of the type and timing of the control tactics that must be used (Forcella, Benech‐Arnold, Sánchez, & Ghersa, 2000). Temperature and water potential have been identified as essential factors that control weed emergence (Forcella et al., 2000). Some indices (Hunter, Glasbey, & Naylor, 1984; Naylor, 1981) and modelling techniques (González‐Andújar, Chantre, Morvillo, Blanco, & Forcella, 2016) are often used to predict weed emergence. In this context, thermal time (TT) models and hydrothermal time (HTT) models are useful tools to describe weed emergence (Bradford, 2002; Grundy, 2003; Zambrano‐Navea, Bastida, & González‐Andújar, 2013). Parametric regression models for emergence are usually employed in this framework. They may offer the simplicity and flexibility required for practical decision support (Grundy, 2003). However, due to the limitations of this approach, different modeling approaches have been proposed, including techniques that account for censoring (Onofri, Gresta, & Tei, 2010; Onofri, Mesgaran, Tei, & Cousens, 2011; Onofri, Piepho, & Kozak, 2019), genetic algorithms (Blanco et al., 2014; Haj Seyed‐Hadi & Gonzalez‐Andujar, 2009), and artificial neural networks (Chantre et al., 2012). Alternatively, the problem of studying the relation between HTT and weed emergence has been dealt with through nonparametric estimation of the distribution and density functions of cumulative HTT (CHTT) at emergence (Cao, Francisco‐Fernández, Anand, Bastida, & González‐Andújar, 2013; Reyes, Francisco‐Fernández, & Cao, 2016). These nonparametric methods have been recently proven to outperform the usual regression approaches in terms of prediction error (González‐Andújar, Francisco‐Fernández, et al., 2016).

In addition, when gathering experimental data, a different problem arises due to the fact that seedlings are generally buried at different depths and, therefore, the best depth at which HTT should be measured has to be selected. For this task, emergence indices have been defined and nonparametric estimators for them have been constructed (Cao, Francisco‐Fernández, Anand, Bastida, & González‐Andújar, 2011).

The techniques required for both, the nonparametric estimation of the density and distribution functions and the emergence indices, have been implemented in the binnednp Rcpp package (Barreiro et al., 2019).

## 2 METHODS

### 2.1 Density and distribution estimation

Let us suppose that modeling the emergence of a certain weed seedling, based on CHTT at emergence, is being investigated. Denote by *n* the number of seedlings that have emerged at the end of the monitoring process, and by *X* the random variable measuring the CHTT at emergence (with density function *f* and distribution function *F*). Since the inspections to count the number of emerged seedlings are performed at a limited number of instants, say *k*, the values *X*_1_, *X*_2_, …, *X_n_*, measuring the CHTT at emergence of every single seedling, cannot be observed. However, what is observed is the total number of seedlings that have emerged in the intervals between consecutive inspection times, *n*_1_, *n*_2_, …, *n_k_*, or the corresponding sample proportions, *w*_1_, *w*_2_, …, *w*_k_, with *w_i_* = *n_i_*/*n*. In this sense, this type of data is called interval‐grouped data.

In this interval‐group framework, if the interest is to estimate the density function *f*, the standard kernel density estimator (Parzen, 1962; Rosenblatt, 1956),

$${\hat{f}}_{h}\left(x\right)=\frac{1}{\mathrm{nh}}\sum_{i=1}^{n}K(\frac{x-X_{i}}{h}),$$

cannot be computed. An appropriate version of this estimator for interval‐grouped data has been proposed in Cao et al. (2011),

(1) $${\hat{f}}_{h}^{g}\left(x\right)=\frac{1}{h}\sum_{i=1}^{k}w_{i}K\left(\frac{x-t_{i}}{h}\right),$$

where *t_i_*, *i *= 1, …, *k*, are the central values between every pair of consecutive observed CHTT. In Equation (1), the function *K*(·) is the kernel function, and *h* is the bandwidth or smoothing parameter, controlling the amount of smoothing.

Similarly, to estimate the distribution function *F*, a kernel distribution estimator adapted for interval‐grouped data, derived from (1), was proposed in Cao et al. (2013),

(2) $${\hat{F}}_{h}^{g}\left(x\right)=\sum_{i=1}^{k}w_{i}K\left(\frac{x-t_{i}}{h}\right),$$

where $K\left(u\right)=\int_{-\infty }^{u}K\left(t\right)dt.$

It has been proven that the selection of the kernel function, *K*(·), is of secondary importance in terms of efficiency. However, the selection of the bandwidth, *h*, is crucial in the behavior of estimators (1) and (2).

As pointed out in the Introduction, nonparametric estimators (1) and (2) are novel approaches to model weed emergence, presenting some advantages over the parametric regression techniques traditionally used in this framework (Cao et al., 2013; González‐Andújar, Francisco‐Fernández, et al., 2016). These estimators, jointly with two types of bandwidth selectors, plug‐in and bootstrap, have been implemented in the binnednp package. Moreover, a new and successful method to select the pilot bandwidth for the bootstrap bandwidths has been proposed and also implemented. Plug‐in bandwidth selectors estimate the unknown terms in the expression of the asymptotically optimal bandwidth, whereas bootstrap bandwidths try to directly estimate the optimal bandwidth by mimicking the sampling process through resampling.

The binnednp package also allows to compute bootstrap confidence bands for the density and distribution functions that can be used to assess the uncertainty of the corresponding estimates. Additionally, the binnednp package includes a function to evaluate the effect of a specific factor on weed emergence, for example, when considering different treatments. This procedure is based on a bootstrap approach properly designed to address the multiple testing problem (Westfall & Young, 1993). The idea of this approach is the following. (a) Split the data set into subsets according to the different levels of the factor under study. (b) Compute the nonparametric estimator of the emergence curve considering the pooled sample and, for each level, the nonparametric estimators of the emergence curves using the corresponding data subsets. (c) A reasonable statistic, *D*, to test the null hypothesis that the factor effect is not significant is defined based on a Cramér‐von Mises distance between the nonparametric estimator with the pooled sample and the nonparametric estimators in the different groups. This distance is inspired in the generalization of the Cramér‐von Mises statistic to the problem of comparing *k* independent samples, proposed by Kiefer (1959). (d) To calibrate the test, a bootstrap procedure is used. For this, under the null hypothesis, *B* resamples for each one of the factor levels are generated, and the corresponding *B* bootstrap statistics, $D_{i}^{\text{∗}}$, *i* = 1, …, *B*, are computed. (e) Finally, given a significance level *α*, the null hypothesis is rejected if *D* is larger than the 1 − *α* quantile of {$D_{1}^{\text{∗}}$, …, $D_{B}^{\text{∗}}$}. The *p*‐value of this test can also be approximated by Monte Carlo. A more detailed description of this algorithm is given in Appendix 3.

Both bandwidth selection methods, plug‐in and bootstrap, for the nonparametric estimators (1) and (2) implemented in the corresponding functions of the binnednp package are described in Appendix 1. Moreover, Appendix 2 contains the steps of the bootstrap algorithm used in the binnednp package to compute confidence bands for the distribution function. Finally, Appendix 3 describes the statistical procedure implemented in the binnednp package to test whether a factor can be statistically significant.

### 2.2 Emergence indices estimation

In this context, another interesting problem is that of finding the best soil depth at which to measure the HTT. For this, moment‐based indices and probability density‐based indices were proposed in Cao et al. (2011), and estimates of them are also implemented in the binnednp package. Some of these index estimators are based on nonparametric methods and require the selection of a bandwidth. Different techniques to automatically obtain approximately optimal bandwidths have also been included in the package.

In order to maximize the predictive power of the weed emergence models considered, one should choose the depth such that the density function of *X*, measuring the CHTT at emergence, is as flatter as possible (or the distribution of *X* has as much spread as possible). Taking this into account, two indices based on the moments of *X*, the coefficient of variation and the kurtosis of *X*, have been considered:

$$I_{1}=\frac{\sigma }{\mu },$$

$$I_{2}=\frac{m_{4}}{\sigma^{4}},$$

where μ=E[X], $\sigma^{2}=V\left[X\right]$, and $m_{4}=E\left[{\left(X-\mu \right)}^{4}\right]$ are the mean, the variance, and the fourth central moment of *X*, respectively. Large values for *I*_1_ and small values for *I*_2_ would be associated with a highly spread and light‐tailed distribution and, therefore, are desirable for good weed emergence prediction properties. Indices based on the density of *X*, namely.

$$J_{1}=\sigma^{3}\int f^{\prime }{\left(x\right)}^{2}dx,$$

$$J_{2}=\sigma^{5}\int f^{\prime \prime }{\left(x\right)}^{2}dx,$$

have been also considered.

The indices *J*_1_ and *J*_2_ measure the curvature of the distribution and density functions of *X*, respectively. Therefore, small values for both *J*_1_ and *J*_2_ are desirable.

## 3 PACKAGE ARCHITECTURE

binnednp is an R package (R Development Core Team, 2019) designed for nonparametric estimation of both density and distribution functions of interval‐grouped data. Although binnednp can be used for the analysis of any variable presented as grouped in intervals, the package and its structure were designed for its use by the weed science research community.

The package was developed using the Rcpp API (Eddelbuettel & François, 2011) which allows the integration of C++ code in R. The parts of the package code relative to bandwidth selection are quite consuming in terms of computation time, especially those that make use of bootstrapping. For this reason, writing portions of the package in C++ was crucial since it allows obtaining numerical results in a very short time. Moreover, the runtime of some of the functions can be further reduced (up to 60%) by means of parallelism with sockets. This was observed in a simulation study (not shown here for the sake of brevity) performed to analyze the CPU time of the functions of the package, when the sample size increases, and the effect of using (or not) parallel computing.

Regarding the structure of the package, it consists of the four functions described below (Sections 3.1, 3.2, 3.3–3.4). A more complete description and additional examples can be found in the reference manual of the package (Barreiro et al., 2019). Next, the following notation is considered in the arguments of those functions:

1. n: Number of seedlings that have emerged at the end of the experiment. In general, it is the size of the unknown complete sample.
2. y: Vector with the measurements of the CHTT at each inspection time. In general, this vector contains the endpoints of the intervals where the data are grouped.
3. w (ni): Vector with the proportion (number) of seedlings that have emerged between each pair of consecutive CHTT. In general, each element of this vector indicates the proportion (number) of observations lying within each of the intervals where the data are grouped.

### 3.1 Density estimation



bw.dens.binned (n, y, w, ni, gboot, pilot.type = 3, hn = 100,
plugin.type = "N", confband = FALSE, alpha = 0.05, B = 1000,
plot = TRUE, print = TRUE, model, parallel = FALSE, pars = new.env())




This function computes the plug‐in and bootstrap bandwidths for the density estimator (1). Regarding the plug‐in bandwidth, with the parameter plugin.type, the iterative process to estimate the bandwidth can be chosen. As for the bootstrap bandwidth, the parameter pilot.type allows the user to select the method to automatically compute the pilot bandwidth needed for the calculation of the bootstrap bandwidth, whereas the parameter gboot allows to manually select that pilot bandwidth. In most situations, it is recommended to employ the default values of these parameters. Additionally, the estimation process can be further personalized using parameters like hn, that determines the number of iterations done during the optimization stage, or B, that indicates the number of bootstrap replicates used for the construction of confidence bands, in case that confband = TRUE. Furthermore, if parallel = TRUE, confidence bands are estimated using parallel computing. Finally, for the sake of comparison with (1), the parameter model allows to fit different parametric families of distributions to the grouped sample. The parameters of these distributions are estimated by maximum likelihood.

### 3.2 Distribution estimation



bw.dist.binned(n, y, w, ni, gplugin, type = "N", confband = FALSE,
B = 1000, alpha = 0.05, plot = TRUE, print = TRUE, model,
parallel = FALSE, pars = new.env())




This function computes the plug‐in bandwidth for the distribution estimator (2). The parameter type allows the user to choose the iterative process to be used to estimate the bandwidth, whereas with the parameter gplugin, the bandwidth used in the last iteration, when type = "A", can be manually selected. Due to the erratic behavior of the bandwidth selector with type = "A", it is strongly recommended to compute the plug‐in bandwidth using type = "N". Anyway, the bootstrap bandwidth selector, computed with the function described below, has shown a better performance than any of the plug‐in bandwidths in most scenarios. If confband = TRUE, bootstrap confidence bands are calculated considering B replicates and using parallel computing, in the case that parallel = TRUE.

Parameter model plays a similar role as in bw.dens.binned.



bw.dist.binned.boot(n, y, w, ni, g, pilot.type = 2, nit = 10,
confband = FALSE, B = 1000, alpha = 0.05, print = TRUE, plot = TRUE,
parallel = FALSE, pars = new.env())




This function computes the bootstrap bandwidth selector for the distribution estimator (2). The parameter pilot.type defines the method to select the pilot bandwidth used for the estimation of the final bandwidth, whereas the parameter g allows the user to manually select that pilot bandwidth. The parameter nit fixes the number of iterations to be done in the optimization stage. If confband = TRUE, bootstrap confidence bands are estimated considering B resamples and using parallel computing, in the case that parallel = TRUE.

### 3.3 Emergence indices estimation



emergence.indices(n, y, w, ni, hseq, hn = 200, nmix = 4, B = 500,
method = "np", last.iter.np = F, confint = FALSE, B.conf = 1000,
alpha = 0.05, print = TRUE, parallel = FALSE, pars = new.env())




This function computes estimates for grouped data of the moment‐based and density‐based emergence indices presented in Section 2.2. In the case of the density‐based indices, with the parameter method, the method to select the bandwidth used for their estimation can be chosen: if method = "plugin", a plug‐in approach is considered, whereas if method = "mix" or method = "np", a parametric or nonparametric bootstrap approach is considered, respectively. If confint = TRUE, bootstrap confidence intervals are constructed considering B resamples and using parallel computing, in the case that parallel = TRUE.

### 3.4 Analysis with different treatments



anv.binned(n, y, trt.w, abs.values = FALSE, B = 500)




This function allows to analyze whether a factor has a significant effect on the emergence curve. The idea behind this approach was briefly explained in Section 2.1, and it is described in detail in Appendix 3. It consists in using a bootstrap approach and a Cramér‐von‐Mises type distance.

In this case, n is a vector composed of the sizes of the complete samples corresponding to each treatment, and trt.w is a matrix each of whose columns contains the proportion of observations lying within each of the intervals for the corresponding treatment. If instead of proportions the user wants to provide absolute values, the parameter abs.values must be set to TRUE. Furthermore, the user can choose the number of bootstrap resamples through the parameter B. The function anv.binned returns the *p*‐value of the test.

## 4 EXAMPLE

In this section, an unpublished data set of wild oat (*Avena sterilis* L.) emergence is considered to illustrate the use of the binnednp Rcpp package. These data were taken from an experiment performed during Winter–Spring 2006–2007 in Gibraleon (37°C 22′N, 6°C 54′W; altitude 26 m), located in the province of Huelva (Andalucia, South of Spain).

Briefly, the experiment consisted in four polyvinylchloride cylinders (250 mm diameter 50 mm height) placed 1 m apart. For each sample, 200 seeds of *A. sterilis* were mixed thoroughly with the soil and distributed over the 0–100 mm depth. Numbers of emerged weed seedlings were recorded once or twice a week and then removed by cutting seedling stems at ground level with minimum disturbance of the substrate. All the data for the cumulative numbers of seedling emergence from the field were converted to a square meter basis. Additionally, following the same procedure as that described in Cao et al. (2011), the CHTT at emergence in the different inspection days, at three depths (10, 20, and 50 mm), was calculated.

The observed emergence data are shown in Table 1. As it can be seen, the cumulative hydrothermal time at emergence cannot be observed for every individual seed, but just in an aggregated way.

**Table 1:** Seedling emergence data of *Avena sterilis*

Date	CHTTT			No. seedlings				Pooled
	Depth			Cylinder				
	10 mm	20 mm	50 mm	1	2	3	4	
27 November 2006	100	92	67	0	0	0	0	0
4 December 2006	160	146	105	0	0	0	0	0
12 December 2006	218	199	143	2	6	8	3	19
14 December 2006	218	217	155	1	0	0	1	2
19 December 2006	218	217	185	2	1	1	3	7
22 December 2006	218	217	199	2	1	1	0	4
26 December 2006	218	217	204	1	1	0	0	2
28 December 2006	218	217	204	0	0	0	0	0
2 January 2007	218	217	204	0	0	0	0	0
5 January 2007	218	217	204	0	2	0	0	2
9 January 2007	218	217	204	2	2	9	2	15
12 January 2007	218	217	204	3	7	18	11	39
18 January 2007	218	217	204	12	7	19	22	60
25 January 2007	218	217	204	6	5	8	13	32
1 February 2007	265	261	232	2	5	7	7	21
9 February 2007	352	340	287	13	12	5	8	38
15 February 2007	405	421	343	7	12	13	4	36
23 February 2007	459	505	421	0	0	1	0	1
5 March 2007	509	571	538	0	0	0	0	0
19 March 2007	509	571	538	0	0	0	0	0
Num emerged seedlings				53	61	90	74	*n* = 278

**Figure 1:**
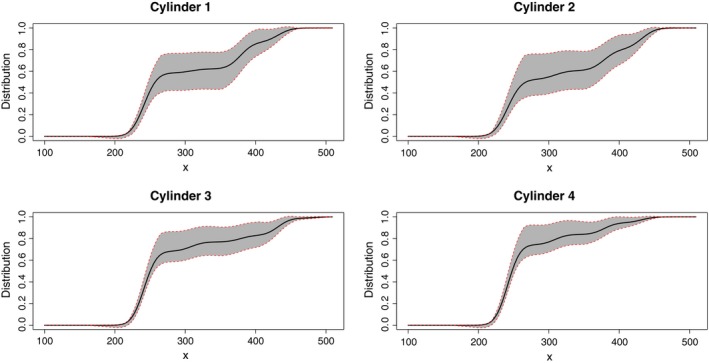
Nonparametric estimates of the emergence curves using the samples in each of the four cylinders, jointly with the corresponding 95% bootstrap confidence bands

In the first part of the study, we use the function anv.binned to evaluate whether a factor (in this case, “the cylinder factor") has a significant effect on the emergence curve, for any of the three depths. As pointed out in Section 2.1, the test implemented in the function anv.binned is based on a Cramér‐von Mises distance between the nonparametric estimator with the pooled sample and the nonparametric estimators in the different levels of the factor. For example, in the case of depth 10 mm, the distance between all these curves could be visually observed comparing Figures 1 and 3. Figure 1 contains the nonparametric emergence curve estimates with bootstrap bandwidths (jointly with the corresponding 95% bootstrap confidence bands) for each one of the four cylinders. Figure 3 depicts the emergence curve estimates using the nonparametric approach with the pooled sample. However, it is clear that a reliable and formal solution for this problem requires the application of a statistical test, such as that implemented in the function anv.binned.

Note that, in this case, the identical experimentation conditions carried out in the four cylinders seem to support the idea that the null hypothesis could be true and, for this, only a very strong evidence against it will lead us to reject the null hypothesis of “nonsignificant cylinder effect."

Denoting by y1, y2, and y3 the CHTT was calculated for 10, 20, and 50 mm, respectively, and after applying the function anv.binned for the three depths, using the following code:



# Observed values of CHTT for each depth
y1 = c(100,160,218,265,352,405,459,509)
y2 = c(92,146,199,217,261,340,421,505,571)
y3 = c(67,105,143,155,185,199,204,232,287,343,421,538)

# size of the complete sample for each treatment
n = c(53,61,90,74)

# nij: number of emerged seedlings for treatment i and depth j
n11 = c(0,0,31,2,13,7,0)
n21 = c(0,0,32,5,12,12,0)
n31 = c(0,0,64,7,5,13,1)
n41 = c(0,0,55,7,8,4,0)

# wij: proportion of emerged seedlings for treatment i and depth j
w11 = n11/n[1]
w21 = n21/n[2]
w31 = n31/n[3]
w41 = n41/n[4]

# Analysis with different treatments for 10 mm
res1 = anv.binned(n,y1,cbind(w11,w21,w31,w41),B=1000)
n12 = c(0,0,2,29,2,13,7,0)
n22 = c(0,0,6,33,5,12,12,0)
n32 = c(0,0,8,56,7,5,13,1)
n42 = c(0,0,3,42,7,8,4,0)

w12 = n12/n[1]
w22 = n22/n[2]
w32 = n32/n[3]
w42 = n42/n[4]

# Analysis with different treatments for 20 mm
res2 = anv.binned(n,y2,cbind(w12,w22,w32,w42),B=1000)

n13 = c(0,0,2,1,2,2,24,2,13,7,0)
n23 = c(0,0,6,0,1,1,24,5,12,12,0)
n33 = c(0,0,8,0,1,1,54,7,5,13,1)
n43 = c(0,0,3,1,3,0,48,7,8,4,0)

w13 = n13/n[1]
w23 = n23/n[2]
w33 = n33/n[3]
w43 = n43/n[4]

# Analysis with different treatments for 50 mm
res3 = anv.binned(n,y3,cbind(w13,w23,w33,w43),B=1000)

the results obtained are:

––––– Result of the application of anv.binned to the samples of the four cylinders –––––



> res1
[1] 0.016

> res2
[1] 0.144

> res3
[1] 0.011




This indicates that, with a significance level of 0.01, the effect of the cylinders is not significant for any of the three depths. The strong certainty of the null hypothesis justifies the use of this significance level (see, e.g. Cramer & Howit, 2004, p. 151, for a comment on the election of the significance level). Therefore, it makes sense to analyze the samples jointly and not separately.

Taking into account the result obtained after applying function anv.binned to the samples of the four cylinders, in the second part of the study, the data with the pooled number of emerged seedlings, given in the last column of Table 1, are considered. Note that the total sample size of emerged seedlings at the end of the experiment is *n* =278.

Given these weed emergence data, a first interesting issue is to find out what is the best depth, among the three possibilities available in this case, 10, 20, and 50 mm, to measure the CHTT in order to have more prediction power. Denoting, as before, by y1, y2, and y3 the CHTT calculated for 10, 20, and 50 mm, respectively, and by ni the vector with pooled number of emerged seedlings, the function emergence.indices can be applied to (y1, ni), (y2, ni), and (y3, ni) to obtain estimates of the indices presented in Section 2.2.



ind1 <- emergence.indices(n, y1, ni) # emergence indices for 10 mm
ind2 <- emergence.indices(n, y2, ni) # emergence indices for 20 mm
ind3 <- emergence.indices(n, y3, ni) # emergence indices for 50 mm

indices <- data.frame(I1=c(ind1$I1,ind2$I1,ind3$I1), I2=c(ind1$I2,ind2$I2,ind3$I2), J1=c(ind1$J1,ind2$J1,ind3$J1), J2=c(ind1$J2,ind2$J2,ind3$J2))




Obtaining the results:

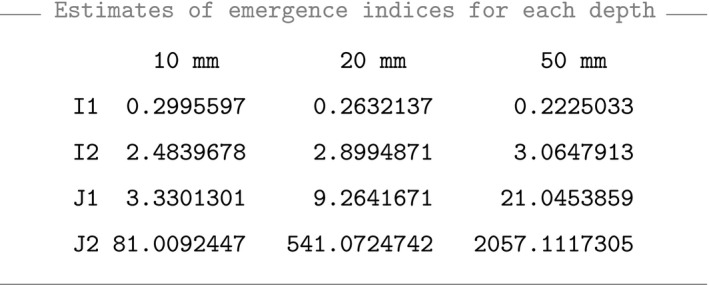

To maximize the predictive power a specific weed emergence model, the HTT should be measured at a depth producing a density function as flat as possible or, equivalently, a distribution function as dispersed as possible. Therefore, small values of *J*_1_ and *J*_2_ are preferable. On the other hand, CHTT samples with higher coefficient of variation (higher value of *I_1_*) and a lower kurtosis (lower value of *I_2_*) will improve weed emergence prediction. Consequently, 10 mm seems to be the best soil depth to predict weed emergence in terms of indices *I*_1_, *I*_2_, *J*_1_, and *J*_2_ and, therefore, only observations at 10 mm are considered in what follows.

Now, the function bw.dens.binned, described in Section 3.1, can be applied using the pooled sample and the CHTT at 10 mm to compute the plug‐in and bootstrap bandwidths for the kernel density estimator (1).



# computing bandwidths for kernel density estimator
dens <– bw.dens.binned (n, y, ni, plot = FALSE) # Bandwidths for density




Obtaining the results:

–––––Plug‐in and bootstrap bandwidths for the density estimator–––––



> dens
$h_plugin
[1] 14.93051
$h_boot
[1] 19.68239




**Figure 2:**
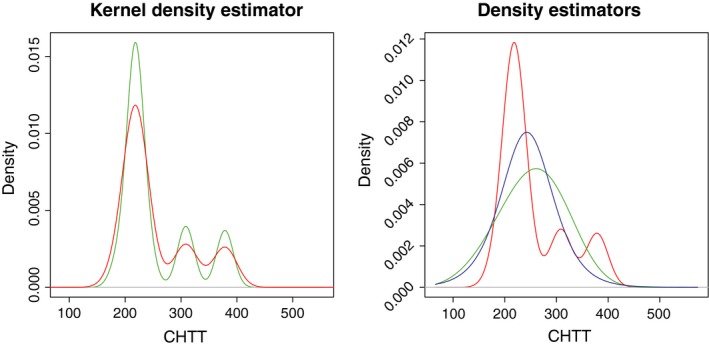
Left panel: kernel density estimates considering plug‐in (green line) and bootstrap (red line) bandwidths. Right panel: parametric Weibull (green line) and logistic (blue line) density estimates, and nonparametric kernel density estimate using the bootstrap bandwidth (red line)

Figure 2 shows, in the left panel, the kernel density estimates computed using (1) with the obtained plug‐in (green lines) and bootstrap (red lines) bandwidths. Moreover, the parameter model was used in bw.dens.binned to fit parametric logistic and Weibull densities to the emergence data. The right panel of Figure 2 shows the corresponding density estimators (green and blue lines for the Weibull and the logistic densities, respectively). Additionally, the nonparametric estimator computed with the bootstrap bandwidth is also included in this figure (red line) for the sake of comparison.

Next, using the functions bw.dist.binned and bw.dist.binned.boot, described in Section 3.2, the plug‐in and bootstrap bandwidths for the kernel distribution estimator (2) are calculated.



# computing bandwidths for kernel distribution estimator

# Plug–in bandwidth
dist_pi <– bw. dist. binned (n, y, ni, plot = FALSE) $h # Plug–in

# Bootstrap bandwidth
dist_boot <– bw. dist. binned. boot (n, y, ni, plot = FALSE)$h # Bootstrap




Obtaining the results:

–––––Plug‐in and bootstrap bandwidths for the distribution estimator–––––



> dist_pi$h
[1] 9.833762
> dist_boot$h
[1] 13.73533




**Figure 3:**
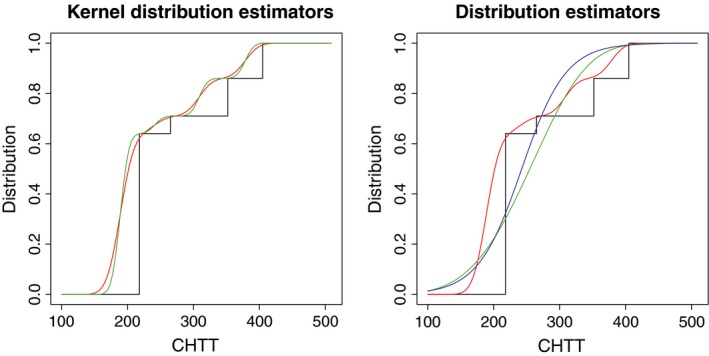
Kernel distribution estimates considering plug‐in (green line) and bootstrap (red line) bandwidths. Right panel: parametric regression fits, Weibull (green line) and logistic (blue line), and nonparametric kernel distribution estimate using the bootstrap bandwidth (red line). The empirical distribution of the grouped sample (black lines) is also shown

Figure 3 shows, in the left panel, the kernel distribution estimates computed using (2) with the obtained plug‐in (green line) and bootstrap (red line) bandwidths, that is, the estimates of the emergence curves using the nonparametric approach. In contrast to the density case, the effect that the bandwidth has on the behavior of the distribution estimator is less evident, since slightly different bandwidths produce very similar distribution estimates. As in the density case, the parameter model in the function bw.dist.binned was set to weibull and logistic to fit parametric regression functions following these models to describe seedling emergence. The corresponding fits are shown in the right panel of Figure 3, using a green line for the Weibull and a blue line for the logistic. The nonparametric distribution estimator (2) is also included in this plot (red line). In both figures, the empirical distribution of the grouped sample data is represented with black lines.

## 5 CONCLUSION

The binnednp R package gives the weed science research community a simple tool to analyze interval‐grouped data. This is useful to study, for example, the CHTT at seedling emergence. Using nonparametric density and distribution estimation, the researcher can both visualize the underlying nature of the data and make predictions without loosing flexibility or making inadequate assumptions about the data. Moreover, estimation of emergence indices measures the adequacy of the depth chosen to register the values of CHTT. Additionally, analyses to test the effect of a specific factor on weed emergence can be also performed.

## CONFLICT OF INTEREST

None declared.

## AUTHOR'S CONTRIBUTIONS

RC, MF‐F, and JLG‐A conceived the ideas and designed the methodology; DB‐U, BBF, RD, and MR wrote the code; JLG‐A provided the data; DB‐U, RC, MF‐F, MR, and JLG‐A analyzed the data. All authors led the writing of the manuscript and contributed critically to the drafts and gave final approval for publication.

## Data Availability

The current stable version of the binnednp package requires R (≥2.10) and is distributed under the GPL‐3 license. It is publicly available on the Comprehensive R Archive Network at https://cran.r-project.org/package=binnednp

## APPENDIX 1

In this appendix, the plug‐in and bootstrap bandwidth selection methods implemented in the functions bw.dens.binned, bw.dist.binned, and bw.dist.binned.boot are briefly described.

### BANDWIDTH SELECTION FOR THE DENSITY FUNCTION

The function bw.dens.binned of the binnednp package computes plug‐in and bootstrap bandwidths for the density estimator (1). Plug‐in bandwidths are obtained minimizing in *h* the expression of the asymptotic mean squared error (AMISE) of (1) and estimating the unknown quantities in the minimizer of the AMISE. Under suitable assumptions, asymptotic properties of (1) were obtained in Reyes et al. (2016). In that paper, it is obtained that the AMISE of (1) is:

(A1) $$\mathrm{AMISE(}{\hat{f}}_{h}^{g})=\frac{1}{4}\mu_{2}{\left(K\right)}^{2}h^{4}A\left(f^{\prime \prime }\right)+\frac{1}{\mathrm{nh}}A\left(K\right),$$

where $\mu_{2}\left(K\right)=\int x^{2}K\left(x\right)dx>0$, $A\left(K\right)=\int K^{2}\left(x\right)dx$ and $A\left(f\prime \prime \right)=\int f^{\prime \prime }{\left(x\right)}^{2}dx.$

From (A1), it follows that the asymptotically optimal global bandwidth for (1) is

(A2) $$h_{g}^{\text{dens}}={\left[\frac{A\left(K\right)}{\mu_{2}{\left(K\right)}^{2}A\left(f^{\prime \prime }\right)n}\right]}^{\frac{1}{5}}.$$

Using (A2) and estimating the unknown quantity *A*(*f*′′), a plug‐in bandwidth for estimator (1) can be derived. In Cao et al. (2011), a nonparametric estimator for *A*(*f*′′), denoted by ${\hat{A}}_{\eta }^{g}$, depending on an auxiliary smoothing parameter η was proposed. Plugging ${\hat{A}}_{\eta }^{g}$ in (A2) gives the plug‐in bandwidth selector for ${\hat{f}}_{h}^{g}$:

$${\hat{h}}_{g}^{\text{dens}}={\left[\frac{A\left(K\right)}{\mu_{2}{\left(K\right)}^{2}{\hat{A}}_{\eta }^{g}n}\right]}^{\frac{1}{5}}.$$

In practice, to calculate ${\hat{A}}_{\eta }^{g}$ a new bandwidth η has to be selected. This pilot smoothing parameter can be selected using again a plug‐in procedure, appearing new auxiliary bandwidths, and so on. The usual strategy is to stop this iterative process after two steps and estimate the unknown quantities assuming that *f* is Gaussian.

As for the bootstrap bandwidth selector for (1) implemented in bw.dens.binned, this is obtained minimizing the bootstrap version of the mean integrated squared error (MISE) of ${\hat{f}}_{h}^{g}$.

Using standard calculations, a closed form expression for the MISE of estimator (1) is given by:

(A3) $$\begin{array}{c} \text{MISE}\left({\hat{f}}_{h}^{g}\right)=E\left[\int {\left({\hat{f}}_{h}^{g}\left(x\right)-f\left(x\right)\right)}^{2}dx\right]\\ =\int {\left[\sum_{i=1}^{k}p_{i}K_{h}\left(x-t_{i}\right)-f\left(x\right)\right]}^{2}dx\\ +\frac{A\left(K\right)}{\mathrm{nh}}-\frac{1}{n}\sum_{i=1}^{k}\sum_{j=1}^{k}p_{i}p_{j}{\left(K\text{∗}K\right)}_{h}\left(t_{i}-t_{j}\right), \end{array}$$

where $K_{h}\left(u\right)=\frac{1}{h}K\left(\frac{u}{h}\right)$, *p_j_* = *F*(*y_j_*) − *F*(*y_j_*_ − 1_), for *j* = 1, …, *k*, and symbol * stands for convolution.

The bootstrap version MISE^*^ of the MISE is obtained as follows. Let

$${\hat{f}}_{\zeta }^{g}\left(x\right)=\sum_{i=1}^{k}w_{i}K_{\zeta }\left(x-t_{i}\right)$$

be the estimator (1) based on a pilot bandwidth ζ. Draw a bootstrap sample $X_{1}^{\text{∗}},\;X_{2}^{\text{∗}},\;\ldots ,\;X_{n}^{\text{∗}}$ from ${\hat{f}}_{\zeta }^{g}$ and, given a bandwidth *h*, consider the analogue of the kernel density estimator, ${\hat{f}}_{h}^{g\text{∗}}\left(x\right)=\sum_{i=1}^{k}w_{i}^{\text{∗}}K_{h}\left(x-t_{i}\right)$, where $w_{i}^{\text{∗}}=F_{n}^{\text{∗}}\left(y_{i}-\right)-F_{n}^{\text{∗}}\left(y_{i-1}-\right)$, with $F_{n}^{\text{∗}}\left(y\right)=\frac{1}{n}\sum_{i=1}^{n}1_{\left\{X_{i}^{\text{∗}}\leq y\right\}}$. Then

(A4) $${\text{MISE}}^{\text{∗}}\left({\hat{f}}_{h}^{g\text{∗}}\right)=E^{\text{∗}}\left[\int {\left({\hat{f}}_{h}^{g\text{∗}}\left(x\right)-{\hat{f}}_{\zeta }^{g}\left(x\right)\right)}^{2}dx\right],$$

where $E^{\text{∗}}$ denotes the bootstrap expectation (with respect to ${\hat{f}}_{\zeta }^{g}$).

Using a parallel process to that followed to obtain (3), it is possible to derive a closed representation for (4). This expression is given by

(A5) $$\begin{array}{c} {\text{MISE}}^{\text{∗}}\left({\hat{f}}_{h}^{g\text{∗}}\right)=\frac{n-1}{n}\sum_{i=1}^{k}\sum_{j=1}^{k}w_{i}^{\zeta }w_{j}^{\zeta }{\left(K\text{∗}K\right)}_{h}\left(t_{i}-t_{j}\right)\\ -2\sum_{i=1}^{k}\sum_{j=1}^{k}w_{i}^{\zeta }w_{j}\left(K_{h}\text{∗}K_{\zeta }\right)\left(t_{i}-t_{j}\right)\\ +\sum_{i=1}^{k}\sum_{j=1}^{k}w_{i}w_{j}{\left(K\text{∗}K\right)}_{\zeta }\left(t_{i}-t_{j}\right)+\frac{A\left(K\right)}{\mathrm{nh}}. \end{array}$$

Note that expression (A5) allows us to directly evaluate MISE* over a grid of values of *h*, without using Monte Carlo. The bootstrap bandwidth $h_{\text{MISE}}^{\text{∗}}$ is obtained minimizing (A5), that is,

$$h_{\text{dens}}^{\text{∗}}=\underset{h>0}{\text{argmin}}{\text{MISE}}^{\text{∗}}\left({\hat{f}}_{h}^{g\text{∗}}\right),$$

To select the pilot bandwidth ζ, the method implemented in the binnednp package is inspired by the idea of smoothing splines, based on selecting the pilot parameter that minimizes the squared distance between the nonparametric density estimator, ${\hat{f}}_{h}^{g}$, and the histogram of the grouped data, $\hat{H}$, plus a penalty term to avoid obtaining very small bandwidths. The idea consists in finding the parameter denoted by $\zeta_{\text{hist}}^{\lambda }$, such that,

$$\zeta_{\text{hist}}^{\lambda }=\;\underset{h>0}{\text{argmin}}\sum_{i=1}^{k}(\hat{H}(t_{i}{)-{\hat{f}}_{h}^{g}\left(t_{i}\right))}^{2}+\lambda \int {\hat{f}}_{h}^{g\prime \prime }{\left(x\right)}^{2}dx,$$

where λ≥0 determines the penalty degree over the global curvature of the nonparametric density estimator, defined in (1). To select an ''optimal'' penalty degree, $\lambda_{\text{opt}}$, we have used the rule of finding the penalty allowing to obtain a pilot bandwidth that best approximate the overall curvature of the population density, that is,

$$\lambda_{\text{opt}}=\underset{\lambda \geq 0}{\text{argmin}}\left|A\left({\hat{f}}_{\zeta_{\text{hist}}^{\lambda }}^{g\prime \prime }\right)-A\left(f^{\prime \prime }\right)\right|.$$

In practice, $\lambda_{\text{opt}}$ can be estimated by

$${\hat{\lambda }}_{\text{opt}}=\underset{{\;}_{\lambda \geq 0}}{\text{argmin}}\left|A\left({\hat{f}}_{\zeta_{\text{hist}}^{\lambda }}^{g\prime \prime }\right)-A\left({\hat{f}}_{\text{mix}}^{\prime \prime }\right)\right|,$$

where ${\hat{f}}_{\text{mix}}$ is a normal mixture model with a maximum number of *r* = 5 components fitted with the grouped‐data sample. In practice, the expectation–maximization (EM) method was used to estimate the parameters of the mixture model, using the BIC criterion to select the best fit.

### BANDWIDTH SELECTION FOR THE DISTRIBUTION FUNCTION

Following analogous arguments to those described for the case of the density function, plug‐in and bootstrap bandwidth selectors can be proposed for the nonparametric distribution estimator given in (2). They are implemented in the functions bw.dist.binned and bw.dist.binned.boot, respectively.

Regarding the plug‐in bandwidth, under some assumptions, it can be obtained that the AMISE of (2) is:

(A6) $$\mathrm{AMISE}\left({\hat{F}}_{h}^{g}\right)=\frac{h^{4}}{4}\mu_{2}{\left(K\right)}^{2}A\left(f^{\prime }\right)+\frac{1}{n}\int F\left(x\right)\left[1-F\left(x\right)\right]dx-\frac{h}{n}C_{0}$$

with $A\left(f^{\prime }\right)=\int f^{\prime }{\left(x\right)}^{2}dx$, and

$$C_{0}=2\int zK\left(z\right)K\left(z\right)dz>0.$$

From equation (A6), it is immediate to get an asymptotically optimal global bandwidth for ${\hat{F}}_{h}^{g}$. Taking the first derivative of (A6), equating to zero and solving for *h*, it is obtained

(A7) $$h_{g}^{\text{dist}}={\left[\frac{C_{0}}{\mu_{2}{\left(K\right)}^{2}A\left(f^{\prime }\right)n}\right]}^{\frac{1}{3}}.$$

In equation (A7), an estimate of *A*(*f*′) is required to have a practical bandwidth. Using a similar process to that described for the case of the density estimator, a practical plug‐in bandwidth selector for ${\hat{F}}_{h}^{g}\left(x\right)$ is obtained:

$${\hat{h}}_{g}^{\text{dist}}={\left[\frac{C_{0}}{\mu_{2}{\left(K\right)}^{2}{\tilde{A}}_{\eta }^{g}n}\right]}^{\frac{1}{3}},$$

where ${\tilde{A}}_{\eta }^{g}$ denotes an estimator of *A*(*f*′) depending on a pilot bandwidthη. A similar iterative procedure to that described in the case of the density function is used here.

On the other hand, the bootstrap bandwidth implemented in the function bw.dist.binned.boot is based on minimizing the bootstrap version of the MISE of ${\hat{F}}_{h}^{g}$.

In this case, using standard calculations and assuming that *F*(*y_k_*) = 1 and *F*(*y*_0_) = 0, it is easy to prove that the expectation and the variance of ${\hat{F}}_{h}^{g}\left(x\right)$ are, respectively,

(A8) $$E\left[{\hat{F}}_{h}^{g}\left(x\right)\right]=\sum_{i=1}^{k}K\left(\frac{x-t_{i}}{h}\right)p_{i}$$

and

(A9) $$\begin{array}{c} V\left[{\hat{F}}_{h}^{g}\left(x\right)\right]=\frac{1}{n}\sum_{i=1}^{k}K^{2}\left(\frac{x-t_{i}}{h}\right)p_{i}\left(1-p_{i}\right)\\ -\frac{2}{n}\sum_{i<j}K\left(\frac{x-t_{i}}{h}\right)K\left(\frac{x-t_{j}}{h}\right)p_{i}p_{j}. \end{array}$$

From (A8) and (A9), it is straightforward to obtain a closed expression for the MISE of the estimator defined in (2):

$$\text{MISE}\left({\hat{F}}_{h}^{g}\right)=E\left\{\int {\left[{\hat{F}}_{h}^{g}\left(x\right)-F\left(x\right)\right]}^{2}dx\right\}=B+V,$$

where,

$$B=\int {\left\{E\left[{\hat{F}}_{h}^{g}\left(x\right)\right]-F\left(x\right)\right\}}^{2}dx$$

denotes the integrated squared bias and

$$V=\int V\left[{\hat{F}}_{h}^{g}\left(x\right)\right]dx$$

is the integrated variance.

To build a bootstrap version of MISE, we consider a pilot bandwidth, ζ, and construct the grouped‐data smooth estimator of *F* as defined in (2), but replacing *h* by ζ. The idea is to draw resamples from ${\hat{F}}_{\zeta }^{g}$, to group the data and to compute the estimator ${\hat{F}}_{h}^{g}$ with those bootstrap samples. The bootstrap resampling plan proceeds as follows.

1. Fix some pilot bandwidth, ζ, and consider the grouped‐data smooth cdf estimator, ${\hat{F}}_{\zeta }^{g}$.
2. Draw $\left(n_{1}^{\text{∗}},\ldots ,n_{k}^{\text{∗}}\right)$ from a multinomial distribution $M_{k}\left(n;{\overset{\sim }{p}}_{1}^{\zeta },\ldots ,{\overset{\sim }{p}}_{k}^{\zeta }\right)$ with p~iζ=F^ζg(yi)-F^ζg(yi-1),i=1,⋯,k, and define $w_{i}^{\text{∗}}=n_{i}^{\text{∗}}/n$.
3. Compute the grouped‐data smooth cdf estimator based on this bootstrap resample:
  $${\hat{F}}_{h}^{g\text{∗}}\left(x\right)=\sum_{i=1}^{k}w_{i}^{\text{∗}}K\left(\frac{x-t_{i}}{h}\right).$$
4. Define the bootstrap version of MISE
  $${\text{MISE}}^{\text{∗}}\left({\hat{F}}_{h}^{g\text{∗}}\right)=E^{\text{∗}}\left\{\int {\left[{\hat{F}}_{h}^{g\text{∗}}\left(x\right)-{\hat{F}}_{\zeta }^{g}\left(x\right)\right]}^{2}dx\right\},$$

where, $E^{\text{∗}}$ denotes the bootstrap expectation (with respect to ${\hat{F}}_{\zeta }^{g}$).

Therefore, defining

p^iζ=p~iζ∑j=1kp~jζ,i=1,2,…,k,

and substituting *p_i_* by p^iζ in (A8) and (A9), the bootstrap version of the MISE admits the following closed expression:

MISE∗F^hg∗=E∗{∫F^hg∗(x)-F^ζgx2dx}=B∗+V∗,

where

$$B^{\text{∗}}=\int \;{\left\{E^{\text{∗}}\left[{\hat{F}}_{h}^{g\text{∗}}\left(x\right)\right]-{\hat{F}}_{\zeta }^{g}\left(x\right)\right\}}^{2}dx$$

and

$$V^{\text{∗}}=\int V^{\text{∗}}\left[{\hat{F}}_{h}^{g\text{∗}}\left(x\right)\right]dx,$$

with

$$E^{\text{∗}}\left[{\hat{F}}_{h}^{g\text{∗}}\left(x\right)\right]=\sum_{i=1}^{k}K\left(\frac{x-t_{i}}{h}\right){\hat{p}}_{i}^{\zeta }$$

and

$$\begin{array}{c} V^{\text{∗}}\left[{\hat{F}}_{h}^{g\text{∗}}\left(x\right)\right]=\frac{1}{n}\sum_{i=1}^{k}K^{2}\left(\frac{x-t_{i}}{h}\right){\hat{p}}_{i}^{\zeta }\left(1-{\hat{p}}_{i}^{\zeta }\right)\\ -\frac{2}{n}\sum_{i<j}K\left(\frac{x-t_{i}}{h}\right)K\left(\frac{x-t_{j}}{h}\right){\hat{p}}_{i}^{\zeta }{\hat{p}}_{j}^{\zeta }. \end{array}$$

Finally, the bootstrap bandwidth is defined as the minimizer of ${\text{MISE}}^{\text{∗}}\left({\hat{F}}_{h}^{g\text{∗}}\right)$, in the smoothing parameter, *h*:

$$h_{\text{dist}}^{\text{∗}}=\;\underset{h>0}{\text{argmin}}{\text{MISE}}^{\text{∗}}\left({\hat{F}}_{h}^{g\text{∗}}\right).$$

As in the case of the bootstrap bandwidth selector for the density function, an important step in this bootstrap procedure is that of selecting the pilot bandwidth ζ. The same type of procedure inspired by the idea of smoothing splines, but adapted for the distribution function, was employed. In this case, the idea consists in finding the parameter, denoted by $\zeta_{\text{emp}}^{\lambda }$, such that,

$$\zeta_{\text{emp}}^{\lambda }=\underset{h>0}{\text{argmin}}\;\sum_{i=0}^{k}{\left[F_{n}\left(y_{i}\right)-{\hat{F}}_{h}^{g}\left(y_{i}\right)\right]}^{2}+\lambda \int {\hat{f}}_{h}^{g\prime }{\left(x\right)}^{2}dx,$$

where λ≥0 determines the penalty degree over the global slope of the nonparametric density estimator, defined in (2). To select an “optimal” penalty degree, $\lambda_{\text{opt}}$, we have used the rule of finding the penalty allowing to obtain a pilot bandwidth that best approximate the overall slope of the population density, that is,

$$\lambda_{\text{opt}}=\underset{\lambda \geq 0}{\text{argmin}}\;\left|A\left({\hat{f}}_{\zeta_{\text{emp}}^{\lambda }}^{g\prime }\right)-A\left(f^{\prime }\right)\right|.$$

In practice, $\lambda_{\text{opt}}$ can be estimated by

$${\hat{\lambda }}_{\text{opt}}=\underset{\lambda \geq 0}{\text{argmin}}\left|A\left({\hat{f}}_{\zeta_{\text{emp}}^{\lambda }}^{g\prime }\right)-A\left({\hat{f}}_{\theta }^{\prime }\right)\right|,$$

where ${\hat{f}}_{\theta }^{\prime }$ represents a parametric estimator of the first derivative of the density function, fitted with the grouped‐data sample and flexible enough to capture, at least partially, the global slope of *f*. It was checked that fitting normal mixture models with a maximum number of *r* = 5 components provided, in general, very good results. In practice, the expectation‐‐maximization (EM) method was used to estimate the parameters of these models, using the BIC criterion to select the best fit.

## APPENDIX 2

As an option, the functions bw.dens.binneC, bw.dist.binned, and bw.dist.binned.boot allow to compute bootstrap confidence bands for the density and distribution functions, using the nonparametric estimators (1) and (2). This bands are designed to contain the whole function with a prescribed high probability, typically 95%, and can be employed, for example, to assess the uncertainty of the corresponding estimates or to perform analyses with different treatments. The procedures used for the density and the distribution functions follow the same steps, and therefore, for the sake of brevity, in this appendix, only the algorithm used in bw.dist.binned and bw.dist.binned.boot to compute the bootstrap confidence bands for the distribution function, *F*(*t*), is detailed.

First, given an initial confidence level, 1–α, for a small α (α = .01 or .05, typically), we start by constructing individual (1–α)‐confidence intervals, ($\ell_{j}$, *u_j_*), for *F*, in the cumulative observed HTT at inspections, *y*_1_, *y*_2_, …, *y_k_*. The process is the following:

1. Fix a pilot bandwidth, *g*, and compute ${\hat{F}}_{g}\left(x\right)$, the smooth cumulative distribution function estimation given in (2).
2. Draw *B* complete bootstrap resamples of size *n* from ${\hat{F}}_{g}\left(x\right):X_{1}^{\text{∗}(j)},X_{2}^{\text{∗}(j)},\ldots ,X_{n}^{\text{∗}(j)},$ *j* = 1, 2, …, *B*. To do this, we first draw *n* observations from a discrete random variable, *I*, that takes the values 1, 2, …, *k*, with probabilities *w*_1_, *w*_2_, …, *w_k_*. Let us denote these observations by $I_{1}^{\left(j\right)},I_{2}^{\left(j\right)},\ldots ,I_{n}^{\left(j\right)}$. Now, for every *i* = 1, 2, …, *n*, we generate $V_{i}^{\left(j\right)}$ with cdf K and define the bootstrap observations as $X_{i}^{\text{∗}(j)}=t_{I_{j}^{\left(j\right)}}+g\cdot V_{i}^{\left(j\right)}.$
3. Consider the incomplete version of these bootstrap resamples: $F_{n}^{\text{∗}(j)}\left(y_{0}\right)\leq F_{n}^{\text{∗}(j)}\left(y_{1}\right)\leq \cdots \leq F_{n}^{\text{∗}(j)}\left(y_{k}\right)$ for *j *= 1, 2, …, B.
4. Compute the estimation given in (2) using the incomplete bootstrap resamples: ${\hat{F}}_{h}^{\text{∗}(j)}\left(x\right)$, *j* = 1, 2, …, *B*.
5. Aproximate the sampling distribution of the stochastic process $D_{n}\left(x\right)={\hat{F}}_{h}\left(x\right)-F\left(x\right)$ by the bootstrap distribution of $D_{n}^{\text{∗}}\left(x\right)={\hat{F}}_{h}^{\text{∗}}\left(x\right)-{\hat{F}}_{g}\left(x\right)$.
6. Now, the $\frac{\alpha }{2}$ and $1-\frac{\alpha }{2}$ quantiles of the bootstrap distribution are computed: $D_{n}^{\text{∗}\left(\left\lceil \frac{\alpha }{2}B\right\rceil \right)}\left(x\right)$ and $D_{n}^{\text{∗}\left(\left\lceil \left(1-\frac{\alpha }{2}\right)B\right\rceil \right)}\left(x\right)(\left\lceil \zeta \right\rceil$ denotes the integer part of ζ).
7. The final bootstrap confidence interval for *F*(*x*) is

$$\left[{\hat{F}}_{h}\left(x\right)-D_{n}^{\text{∗}\left(\left\lceil \left(1-\frac{\alpha }{2}\right)B\right\rceil \right)}\left(x\right),{\hat{F}}_{h}\left(x\right)-D_{n}^{\text{∗}\left(\left\lceil \frac{\alpha }{2}B\right\rceil \right)}\left(x\right)\right].$$

Steps 4–7 are repeated for every *x* = *y_j_*, *j* = 1, …, *k*, and, therefore, *k* pointwise intervals ($\ell_{j}$, *u_j_*), for each value *F*(*y_j_*), *j* = 1, …, *k*, are calculated. Individual confidence intervals have approximately the nominal coverage probability, 1–*α*, when they are considered separately (for a particular grid point). However, the probability that the whole curve is included in the band depicted by the set of intervals is much smaller. This is known as the multiple range testing problem or the false discovery rate in high dimensional statistical problems.

A typical way to correct for multiple testing is the popular Bonferroni approach. In a hypothesis testing context, the idea behind this approach is to consider a new significance level, *α*_Bonf _= *α*/*k*, and compute individual tests using this new level. The resulting multiple test has a multiple level which is much closer to the desired *α*. However, it is well known that the Bonferroni approach is a conservative procedure. In our context, this means that the joint coverage probability of the confidence "band," computed with the Bonferroni approach, would be larger than the desired 1*–α*.

Starting from the conservative Bonferroni approach and the anticonservative individual testing approach, the following algorithm finds an approximate (1–*α*) confidence interval, with a given approximation error *δ* (*δ* is typically small in comparison with the nominal *α*, for instance $\delta =\frac{\alpha }{10}$):

1. Fix $\alpha_{\text{low}}^{\left(0\right)}=\alpha_{\text{Bonf}}=\frac{\alpha }{k}$ and $\alpha_{\text{high}}^{\left(0\right)}=\alpha$. Fix the iteration number, *i* = 0.
2. Compute $\alpha_{\text{mean}}^{\left(i\right)}=\frac{\alpha_{\text{low}}^{\left(i\right)}+\alpha_{\text{high}}^{\left(i\right)}}{2}.$
3. Use the bootstrap resamples to compute individual confidence intervals with $1-\alpha_{\text{low}}^{\left(i\right)},\;1-\alpha_{\text{mean}}^{\left(i\right)}\;\text{and}\;1-\alpha_{\text{high}}^{\left(i\right)}$ confidence levels.
4. Compute, with the same bootstrap resamples, the proportion of bootstrap curves that are included in each of these confidence bands. These proportions satisfy $p_{\text{low}}^{\left(i\right)}\geq p_{\text{mean}}^{\left(i\right)}\geq p_{\text{high}}^{\left(i\right)}\;\text{and}\;p_{\text{low}}^{\left(i\right)}\geq 1-\alpha \geq p_{\text{high}}^{\left(i\right)}.$
5. If $p_{\text{mean}}^{\left(i\right)}\geq 1-\alpha$, then define $\alpha_{\text{low}}^{\left(i+1\right)}=\alpha_{\text{mean}}^{\left(i\right)}$ and $\alpha_{\text{high}}^{\left(i+1\right)}=\alpha_{\text{high}}^{\left(i\right)}$. Otherwise define $\alpha_{\text{low}}^{\left(i+1\right)}=\alpha_{\text{low}}^{\left(i\right)}$ and $\alpha_{\text{high}}^{\left(i+1\right)}=\alpha_{\text{mean}}^{\left(i\right)}.$
6. Stop at step *i* if $\left|p_{\text{mean}}^{\left(i\right)}-\left(1-\alpha \right)\right|<\delta$. Otherwise increase *i* in one unit and repeat Steps 2–5.

The final approximate (1–*α*) simultaneous confidence intervals are those obtained for level $1-\alpha_{\text{mean}}^{\left(i\right)}$ in the last iteration.

## APPENDIX 3

In this appendix, the statistical procedure implemented in the function anv.binned, presented in Section 3.4, is described in detail.

Consider an interval‐grouped sample of size *n*. This means that given a set of *k* intervals [*y*_j–1_, *y_j_*), *j* = 1, …, *k*, only the number of observations (*n*_1_, …, *n_k_*) within each interval (instead of the value of every single observation) is known. Note that *n* = *n*_1_ + … +*n_k_*. For example, as described in Section 2.1, in the weed emergence problem studied in this paper, the vector (*y*_0_, *y*_1_, …, *y_k_*) denotes the CHTT at emergence and the vector (*n*_1_, …, *n_k_*) represents the number of seedlings that have emerged in each interval. Additionally, consider that there exists a factor of interest, with *G* levels or treatments, and we want to test whether this factor has a significant effect in the emergence. Let us denote by $N_{j}^{i}$ the number of observations in the interval *j* considering the treatment *i*, with *j* = 1, …, *k* and *i* = 1, …, *G*. Then, $\sum_{j=1}^{k}N_{j}^{i}=N_{i}$, where *N_i_* denotes the number of observations associated with treatment *i*, *i* = 1, …, *G* (*n* = *N*_1_+⋯ + *N_G_*), and $\sum_{i=1}^{G}N_{j}^{i}=n_{j}$, *j* = 1, …, *k*.

Denoting by *F_i_* the emergence curve considering treatment *i*, *i* = 1, …, *G*, the problem of testing whether the factor has a significant effect in the emergence can be formulated as:

$$\left\{\begin{array}{c} H_{0}:F_{i}=F_{j},\forall i,j\in \left\{1,\ldots ,G\right\}\\ H_{1}:\left.\exists i,j\in \{1,\ldots ,G\}\right|F_{i}\neq F_{j} \end{array}\right.$$

A reasonable statistic to address this hypothesis testing is, for example, the following one, of Cramér‐von Mises type, based on the statistic to compare *k* independent samples, proposed by Kiefer (1959):

$$D=\sum_{i=1}^{G}N_{i}\int {\left({\hat{F}}_{i}^{g}\left(t\right)-{\hat{F}}^{g}\left(t\right)\right)}^{2}d{\hat{F}}^{g}\left(t\right),$$

where ${\hat{F}}_{i}^{g}\left(\cdot \right)$ denotes the corresponding nonparametric estimator of $F_{i}\left(\cdot \right)$, using (2), computed using *N_i_* and ($N_{1}^{i},\ldots ,N_{k}^{i}$), for every *i* = 1, …, *G*, and where ${\hat{F}}^{g}\left(\cdot \right)$ represents the nonparametric estimator (2) using the pooled sample *n* and (*n*_1_, …, *n_k_*).

Intuitively, the null hypothesis *H*_0_ will be accepted for small values of *D* and rejected for large values of *D*. To calibrate the test, a bootstrap procedure is employed to approximate the sampling distribution of *D*. The specific steps are the following:

1. Using a bandwidth *h* (e.g., the one provided by bw.dist.binned.boot), consider the grouped‐data nonparametric estimator, ${\hat{F}}_{h}^{g}$, using the pooled data set with *n* and (*n*_1_, …, *n_k_*).
2. For each treatment *i*, *i* = 1, …, *G*, draw ($N_{1}^{i\text{∗}},\ldots ,N_{k}^{i\text{∗}}$) from a multinomial distribution $M_{k}\left(N_{i};{\hat{p}}_{1}^{h},\ldots ,{\hat{p}}_{k}^{h}\right)$, with ${\hat{p}}_{j}^{h}={\hat{F}}_{h}^{g}\left(y_{j}\right)-{\hat{F}}_{h}^{g}\left(y_{j-1}\right)$, *j* = 1, …, *k*, and define $w_{j}^{i\text{∗}}=N_{j}^{i\;\text{∗}}/N_{i}$.
3. Using the weights $w_{1}^{i\;\text{∗}},\ldots ,w_{k}^{i\;\text{∗}}$, compute the grouped‐data nonparametric estimator ${\hat{F}}_{i}^{g\;\text{∗}}$, for each treatment *i* = 1, …, *G*, and the nonparametric estimator ${\hat{F}}^{g\;\text{∗}}$ using the pooled bootstrap resample *n* and $\left(\sum_{i=1}^{G}N_{1}^{i\;\text{∗}},\ldots ,\sum_{i=1}^{G}N_{k}^{i\;\text{∗}}\right)$. In all the cases, the bootstrap bandwidths obtained with bw.dist.binned.boot can be employed.
4. Define the bootstrap version of *D*:
  $$D^{\text{∗}}=\sum_{i=1}^{G}N_{i}\int {\left({\hat{F}}_{i}^{g\;\text{∗}}\left(t\right)-{\hat{F}}^{g\;\text{∗}}\left(t\right)\right)}^{2}d{\hat{F}}^{g\;\text{∗}}\left(t\right),$$
5. Steps 2–4 are repeated a large number of times, *B* and, then, a sequence {$D_{1}^{\text{∗}},\ldots ,D_{B}^{\text{∗}}$} is obtained. Given a significance level *α*, the null hypothesis is rejected if
  $$D>D_{\left(\left\lceil (1-\alpha )\cdot B\right\rceil \right)}^{\text{∗}},$$

where $\left\lceil \cdot \right\rceil$ represents the integer part, and ${\left\{D_{\left(i\right)}^{\text{∗}}\right\}}_{i=1}^{B}$ is the sample ${\left\{D_{i}^{\text{∗}}\right\}}_{i=1}^{B}$ arranged in increasing order of magnitude. Additionally, the *p*‐value of this test can be approximated by:

$$\hat{p}=\frac{1}{B}\sum_{i=1}^{B}I_{\left\{D_{i}^{\text{∗}}>D\right\}},$$

where $I_{\left\{\cdot \right\}}$ is the indicator function.

It is important to note that with the resampling process described in Step 2, the bootstrap resamples for each treatment are generated under the null hypothesis.
